# Expression of FOXO6 is Associated With Oxidative Stress Level and Predicts the Prognosis in Hepatocellular Cancer: A Comparative Study

**DOI:** 10.1097/MD.0000000000003708

**Published:** 2016-05-27

**Authors:** Hai-Yong Chen, Yao-Min Chen, Jian Wu, Fu-Chun Yang, Zhen Lv, Xiao-Feng Xu, Shu-Sen Zheng

**Affiliations:** From the Division of Hepatobiliary and Pancreatic Surgery, Department of Surgery, First Affiliated Hospital, School of Medicine, Zhejiang University,Key Laboratory of Combined Multi-organ Transplantation, Ministry of Public Health, Key Laboratory of Organ Transplantation, ZheJiang Province, Hang Zhou 310003,China; Collaborative Innovation Center for Diagnosis and Treatment of Infectious Diseases (H-YC, Y-MC, J-W, F-CY, Z-L, X-FX, S-SZ).

## Abstract

The aim of this study was to explore the association of Forkhead box O6 (FOXO6) expression with oxidative stress level and prognosis of hepatocellular cancer (HCC).

The case group included tissues of HCC from 128 patients who were hospitalized in Division of Hepatobiliary and Pancreatic Surgery, Department of Surgery of First Affiliated Hospital, School of Medicine, Zhejiang University. The control group included normal liver tissues from 74 patients. RT-PCR and Western blot were used to test expressions of FOXO6, heme oxygenase (HO)-1, glutathione peroxidase (GPx), superoxide dismutase (SOD), and catalase (CAT). Dihydroethidium (DHE) was dyed to observe reactive oxygen species (ROS) level. Immunohistochemistry was used to test FOXO6 expression. FOXO6 was silenced in HepG2 cells to detect cell proliferation and apoptosis. The expressions of ROS, HO-1, GPx, SOD, CAT, p27, and cyclin D1 were also detected to further explore the possible mechanism.

The expressions of FOXO6, HO-1, GPx, SOD, and CAT in HCC tissue was significantly higher than those in normal and adjacent HCC tissues (*P* <0.05). The tumor size, TNM stage, Alpha-fetoprotein **(**AFP) level, the presence or absence of hepatitis B surface antigen (HbsAg), and differentiation degree were related to FOXO6 expression level (all *P* <0.05). COX analysis showed that high FOXO6 expression, male, positive HBsAg, advanced TNM staging, high expression of AFP, and low degree of differentiation were all risk factors for prognosis in HCC (*P* <0.05). Compared with the blank group (C group, without transfection) and the negative control (NC) group, the mRNA expressions of ROS, FOXO6, HO-1, SOD, GPx, and CAT were decreased (*P* <0.05). si-RNA group had significantly decreased proliferation speed during 24 to 72 hours (*P* <0.05), whereas si-FOXO6 group had remarkably increased G0/G1 staged cells and decreased S-staged cells (*P* <0.05). The si-FOXO6 group showed notably increased apoptosis rate (*P* <0.05) and p27 expressions as well as decreased cyclin D1 expressions (*P* <0.05).

FOXO6 was highly expressed in HCC tissue and was related to oxidative stress levels. Furthermore, FOXO6 expression can be used as a biomarker for deterioration and prognosis of liver cancer, which may provide a novel treatment target for HCC therapy.

## INTRODUCTION

Hepatocellular cancer (HCC) is a malignant tumor, causing ∼1 million deaths worldwide annually.^1,2^ It is reported that surgical treatment, transcatheter arterial chemoembolization (TACE), portal vein chemoembolization (PVCE), and radiotherapy are effective treatments for malignant tumors; however, the present trend in HCC treatment is comprehensive utilization of these means.^3^ Although treatments for HCC have developed rapidly, its survival rate is still relatively low and because of the vague early symptoms of HCC, the majority of cases is in intermediate stage or advanced stage at the initial diagnosis.^4^ The early treatment of HCC contributes to a better prognosis, therefore, the early diagnosis and prognostic marker screening can improve diagnostic effect, increase survival rate of patients, and improve their life quality.^5^ The presently found tumor markers for treatment of primary HCC include alpha-fetoprotein (AFP), des-gamma-carboxy prothrombin (DCP), and TIP30 protein.^6–8^

Forkhead box (FOXO) gene family, comprising >100 members, is evolutionarily conserved in the human genome with a similar sequence among its members and most of its mediated reactions are related to the insulin/P13K/AKT signaling pathway; the functions of its target gene include apoptosis, oxidative stress, glucose metabolism, cell differentiation, anti-aging, and tumor suppression.^9^ The presently found FOXO gene families in mammal include FOXO1, FOXO3, FOXO4, and FOXO6; moreover, FOXO6 was found to play an important role in oxidative stress of cell proliferation.^10,11^ Oxidative stress generally refers to a process in which imbalance of oxygen free radical (OFR) occurs in the process of production and clearance, and reactive OFR accumulates in body resulting in oxidative damage.^12^ The occurrence of HCC is a complex interaction which involves in many genes and needs cooperation of many steps, whereas oxidative stress plays an important role in the occurrence and development of this disease.^13^ Based on the association of FOXO6 with oxidative stress, it is reasonable to hypothesize that FOXO6 may be implicated in the disease development. However, there are no reports about relationship of FOXO6, oxidative stress level, and prognosis of HCC presently. The study aims to explore the relationship between FOXO6 expression and oxidative stress as well as the relationship between FOXO6 expression and clinicopathological features of HCC, to search for potential risk factors in the formation of HCC to provide a new direction for treatment and prognosis of HCC.

## MATERIALS AND METHODS

### Subjects

The case group included 128 HCC patients who received liver tumorectomy and were hospitalized in Division of Hepatobiliary and Pancreatic Surgery, Department of Surgery of the First Affiliated Hospital, School of Medicine, Zhejiang University from January 2008 to April 2010. At the meantime, 74 cases of normal hepatic tissues from liver trauma or hepatic hemangioma were excluded from any hepatic disease by clinical examination, laboratory, and imageological examination, thus obtained as the control group. All HCC tissue samples and normal hepatic tissues were obtained after surgical operation and were immediately kept in liquid nitrogen till further usage.^14^ The procedures for sample collection were in accordant with the protocols and declarations of our hospital. The patients’ age ranged from 20 to 72 years (mean age 48.36 ± 9.86). All of the patients had complete medical records. All included patients had complete medical data and pathological results of paraffin-embedded specimen were reviewed by the 1 pathological doctor. All of the patients did not get any preoperative chemotherapy. The informed consents were provided and the experiment was approved by the ethical committee of First Affiliated Hospital, School of Medicine, Zhejiang University.

### Dihydroethidium Dyeing to Observe Reactive Oxygen Species Level in Liver Tissues^15^

Reactive oxygen species (ROS) fluorescent probe-dihydroethidium (DHE) was a classic method to test ROS level in tissues or cells. DHE could enter into cells through living cell membrane freely, then it was oxidized by ROS to form oxidized ethidium which could mix with chromosomal DNA to produce red fluorescence. According to kit brochures (Vigorous Biotechnology, Beijing, China), firstly dilute probe solution to the desired concentration, replace tissue perfusion fluid with staining solution, incubate for 10 to 90 minutes at room temperature without light exposure, and then clean the tissues with fresh solution. Subsequently, the samples were observed under a fluorescence microscope, motivated by blue light or green light, and photographed the red image of cells. ROS-positive cells were stained red in the whole nucleus and fluorescence intensity could represent the ROS level.

### Immunohistochemistry

All tissue samples were fixed by formalin, embedded by paraffin and then sliced into tissue sections (3 μm). After the sections were repaired by xylene, gradient alcohol, and citrate antigen repair fluid (pH 7.2–7.4), the sections were added with primary antibody for FOXO6 and then incubated overnight at 4°C. Subsequently, the samples were incubated for 1 hour at 37°C. After washing the sections with 0.01 mol/L phosphate buffer saline (PBS), we dropped biotin-labeled working liquid (Wuhan BOSTER Company) as a secondary antibody. They were incubated for 30 minutes at 37°C. After DAB developed for 10 minutes, samples were dyed with hematoxylin, they were mounted with alcohol gradient dehydration and neutral gum. A microscope was used for observation.

The criteria of experimental results are as follows: immunohistochemical-positive reactant showed brown yellow staining-positive cell in nucleus. According to the percentage of positive area in total slice area: 0 point for 0%, 1 point for 1% to 10%, 2 points for 11% to 50% and 3 points for >50%. Staining intensity were scored and stratified as follows: 0 point for negative, 1 point for weak positive, 2 points for middle positive, and 3 points for strong positive. If the sum of the above 2 scores was ≥4 points, it was defined as high expression; if the sum is <4 points, it was defined as low expression.^16^

### Western Blot

Protein was extracted from all tissues. According to BCA kit instructions (BioTeke Corporation, China), protein concentration was measured and loading buffer (30 μg/well) was added into the extracted protein, followed by boiling for 10 minutes at 95°C. Polyacrylamide gel electrophoresis (10%) was used to isolate protein. It rotated wet with the electrophoretic voltage transferred from 80 to 120 V and then changed to transmembrane voltage 100 mV for 45 to 70 minutes. After polyvinylidene fluoride (PVDF) transferred membrane and sealed with 5% bovine serum albumin (BSA) for 1 hour at room temperature, the extracted protein was incubated with different primary antibodies respectively overnight at 4°C. After being rinsed with TRIS-buffered saline-Tween (TBST) 3 times every 5 minues, the extracted protein was incubated with corresponding secondary antibodies for 1 hour at room temperature. We washed membrane 3 times every 5 minutes and used chemiluminescence reagents to develop. β-actin acted as internal reference. Bio-rad Gel Doc EZ imager was used to develop. Image J software was used to record gray value of target band and grayness ratio of target band and internal reference was calculated. Experiments were performed by triplicates.

### Reverse Transcription-Polymerase Chain Reaction (RT-PCR)^17^

Based on gene sequence published by Genbank database, Primer 5.0 primer-design software was used to design primer as presented in Table 1. According to NucleoSpin1RNA II kit introduction (Macherey-Nagel, Germany), the total ribose nucleic acids (RNAs) of HCC tissues, adjacent cancer tissues, and normal tissues were extracted and absorbance (OD) at 260/280 wavelength was recorded using an ultraviolet spectrophotometer to calculate RNA concentration. Then RNA was transcribed into complementary deoxyribonucleic acid (cDNA) according to kit introduction (Invitrogen GmbH, Karlsruhe, Germany). PCR system contains a total of 20 μL: 10 μL of 2 × super Real, 0.6 μL of forward primer and reverse primer, respectively, 4 μL of reverse transcription product, 2 μL of 50 × ROX, 2.8 μL of RNase-free water. PCR was 2-step reaction conditions: initial denaturation at 95^o^C for 15 minutes, followed by 40 cycles of denaturation at 95^o^C for 10 seconds, annealing for 30 seconds, and extension for 30 seconds. β-actin acted as internal reference. Solubility curve was used to evaluate the reliability of PCR results, and CT value (amplify inflection point of power curve) was obtained to calculate the relative expression of target gene according to 2^-ΔΔ^Ct. Experiments were performed by triplicates.

**TABLE 1:**
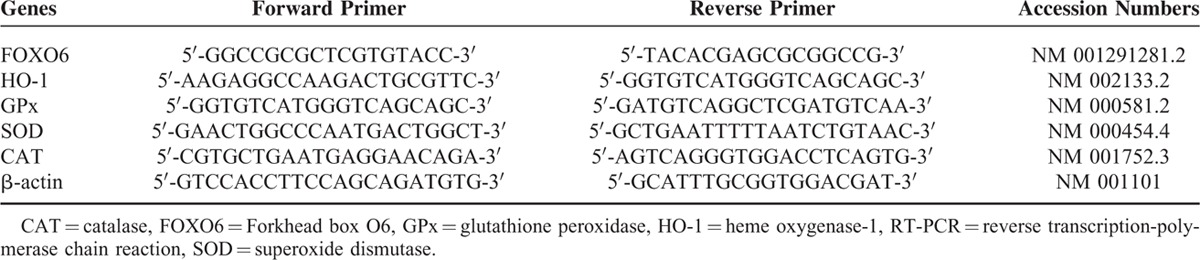
Primer Design of RT-PCR

### Follow-Up

The follow-up started once patients finished the systemic treatments and was discharged from hospital and it ended in April 2015. If patients survived until the observation ended, the censored data was processed. For patients lost to follow-up, the last statistics data was processed. Follow-up was recorded through outpatient data sources, telephone, or medical record review. After 5 years of follow-up, 14 patients were lost to follow-up with lost follow-up rate of 10.94%. Survival time was calculated from month of presentation to month of death. Overall survival (OS) was defined as the duration from HCC resection to patients’ death caused by any reason. Disease-free survival (DFS) was defined as the duration from HCC resection to patients’ death caused by HCC recurrence or progress.

### Cell Culture

Hepatocyte cell line L02 and hepatoma cell line HepG2, SMMC7721, and Huh7 were purchased from Chinese Academy of Sciences Cell Bank. Cryogenic vial is rapidly shaken in water bath case at 37°C. The melted cells were centrifuged at 1000 rpm for 3 minutes and then medium were removed. Cells were resuspended with new culture medium in a 25 cm bottle. L02 and SMMC7721 cells were maintained by Roswell Park Memorial Institute (RPMI) 1640 containing 10% fetal calf serum (FBS), whereas HepG2 and Huh7 were cultured with Dulbecco minimum essential medium (DMEM) containing 10% FBS. Cultures were incubated in a humidified atmosphere with 5% CO_2_ at 37°C, supplemented with appropriate concentration of penicillin, streptomycin, and ciprofloxacin (CPFX). The cells were attached to the plate, followed by digestion by 0.25% pancreatin (containing 0.1% EDTA). RPMI 1640 and DMEM were bought from Giboc Co. (Grand Island, NY). The expression of FOXO6 mRNA in 4 groups was assessed by RT-PCR.

### Plasmid Transfection

Six-well cells were divided into 3 groups: blank group (C group: without transfection), negative control group (NC group: transfected with 5′-UUCUCCGAACGUGUCACGUTT-3′), and transfected group (si-FOXO6 group: transfected with 5′-CAUGACUUAGCAUACGAAGUAC-3′ of FOXO6-siRNA). The transfection of HCC line HepG2 was conducted according to the manufacturer's instructions of Lipofectamin2000 (Invitrogen, Carlsbad, CA). Culture fluid for HepG2 cell with a confluence of 50% to 60%, was removed. Then the cells were washed by PBS 2 times, added with 1 mL serum-free medium without double antibody and incubated in an incubator. Sequences mentioned were obtained from Genechem Co., Shanghai, China. The ROS levels and expression of relative peroxiredoxins were detected using RT-PCR.

### Cell Proliferations by CCK-8

All experiments were strictly carried according to the supplier's recommendations of Cell Counting Kit-8 (Beyotime Co., Shanghai, China). Single cell suspension was prepared after 48 hours of transfection. Cells were seeded into 96-well plates at a concentration of 200 μL/well (cells number: 5 × 10^3^). Cell Counting Kit-8 (10 μL) was added to each well respectively at 0, 24, 48, and 72 hours post-transfection. OD_450_ was detected at 450 nm by a microplate reader. Growth curve was drawn according to the average value of OD as vertical scale and time (h) as abscissa. Experiments were performed in triplicates.

### Cell Cycle and Apoptosis Analysis by Flow Cytometry

For analysis of cell cycle, cells were collected after 48-hour transfection and washed 1 time with PBS. Fixed by 75% ethyl alcohol at 4°C overnight, cells were washed 3 times with PBS. Cells were suspended in 1 mL PBS for 30 minutes containing 40 μg PI, 100 μg/mL RNase A, and 0.1% TritonX-100. FACS Calibur flow cytometry (BD Biosciences, San Jose, CA) was used to detect cell cycle. Experiments were performed in triplicates.

To assay the cell apoptosis, samples were washed and resuspended after 48-hour transfection. Then cells were marked by FACS Calibur flow cytometry and analyzed by Cell Quest software. Positive Annexin V cells were proved to be dead. Experiments were performed in triplicates.

### Western Blot Analysis

Cells protein were harvested after 48-hour transfection and separated by 10% sodium dodecyl sulfate-polyacrylamide gel electrophoresis (SDS-PAGE). Samples were transferred to the surface of 0.22 μm nitrocellulose membrane (Sigma Co.). After blocked with milk, membranes were incubated for 2 hours and washed by TBST 4 times. Subsequently, the primary antibody was added for overnight. Washed by TBST 4 times (10 min/time), cells were incubated with horse radish peroxidase (HRP)-labeled secondary antibodies at room temperature for 1 hour. Substrates were added for color developing. Samples were analyzed by Auto-radiograms. Glyceraldehyde-3-phosphate dehydrogenase (GAPDH) was considered as internal reference. GAPDH and anti-p27 were purchased from Santa Cruz Biotechnology Co. Anti-cyclin D1 and HRP-labeled secondary antibodies were bought form Cell signaling Technology Co. Both primary antibody and secondary antibody were dealt with TBST and diluted to a concentration of 1: 1000. Experiments were performed in triplicates.

### Statistical Analysis

SPSS18.0 statistical software was used to analyze data. Measurement data was expressed as  
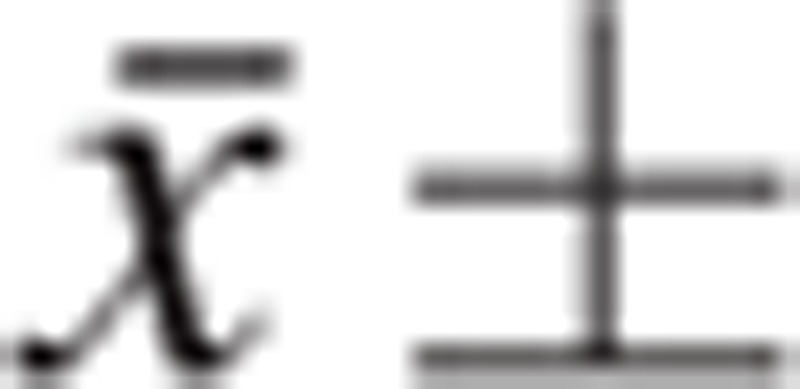
standard deviation (SD). The comparison of 2 groups of measurement data in normal distribution was performed by *t* test and the comparison of many groups was performed by one-way ANOVA analysis. Quantitation data was presented as percentage and ratio and was performed using the chi-square test. As for survival analysis, Kaplan–Meier was used to draw survival curve and used log-rank test for it. For prognosis, we usually used Cox proportional hazards regression analysis of multiple factors. *P* values <0.05 were considered statistically significant.

## RESULTS

### Expression of FOXO6 in Normal Tissue, HCC Tissue, and Adjacent Cancer Tissue

**FIGURE 1:**
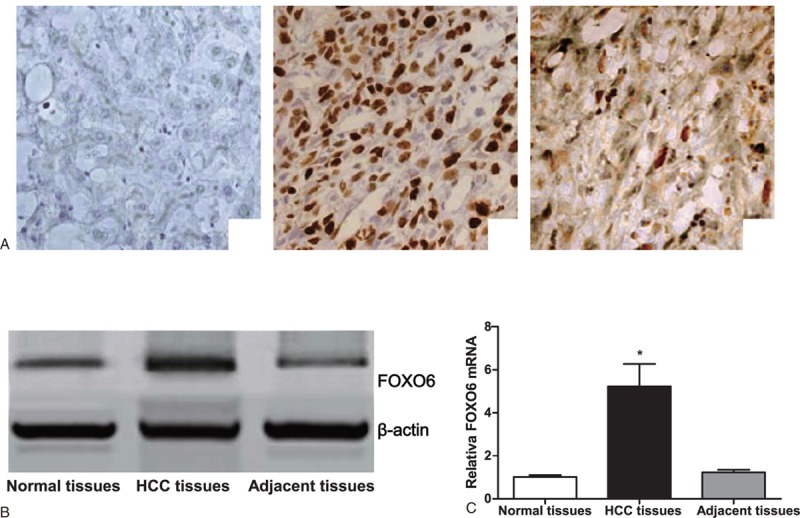
Expression of FOXO6 in normal tissue, HCC tissue, and adjacent cancer tissue. (A) Immunohistochemistry shows expression of FOXO6 in normal tissue (A-a), HCC tissue (A-b), and adjacent cancer tissue (A-c); (B) Western blot shows expression of FOXO6 in normal tissue, HCC tissue, and adjacent cancer tissue; (C) RT-PCR shows expression of FOXO6 mRNA level in normal tissue, HCC tissue, and adjacent cancer tissue. ∗Comparison with normal tissue, *P* <0.05; HCC = hepatocellular carcinoma, FOXO6 = Forkhead box O6, RT-PCR = reverse transcription-polymerase chain reaction.

The immunohistochemistry results showed that FOXO6-positive reactant was presented as brown yellow staining-positive cell in nucleus. The results also found that the expression of FOXO6 in HCC tissue was higher than that in adjacent cancer tissue and normal tissue. The expression of FOXO6 in adjacent cancer tissue was weakly positive (Figure 1A). The results of Western blot demonstrated that the protein expression of FOXO6 in HCC tissue significantly upregulated in comparison with adjacent cancer tissue and normal tissue. The expression of FOXO6 in adjacent cancer tissue was upregulated slightly in comparison with normal tissue (Figure 1B). The results of RT-PCR showed that the expression of FOXO6 mRNA in HCC tissue increased significantly in comparison with adjacent cancer tissue and normal tissue (both *P* <0.05) (Figure 1C).

### Relationship between FOXO6 Expression and ROS Levels

In HCC tissue and adjacent cancer tissue, patients with high FOXO6 expression had a significantly elevated ROS level compared with those with low FOXO6 expression (both *P* <0.05). But in normal tissue, the ROS level had no significant difference between high FOXO6 expression group and low FOXO6 expression group (*P* >0.05) (Table 2).

**TABLE 2:**
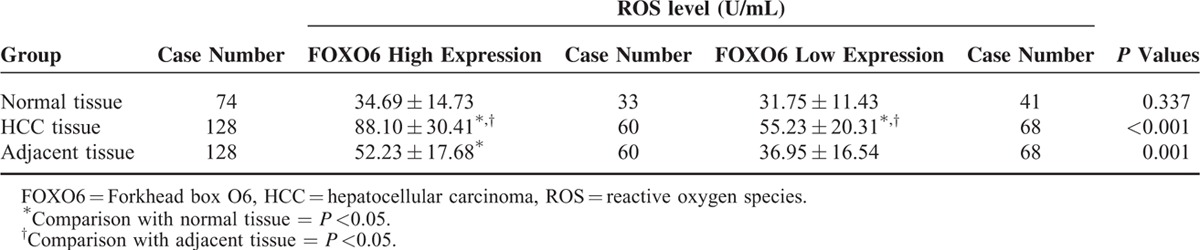
Relationship Between FOXO6 Expression Level and ROS Level in Normal Tissue, HCC Tissue and Adjacent Tissue

### Peroxiredoxins Protein and mRNA Expression in HCC Tissue

**FIGURE 2:**
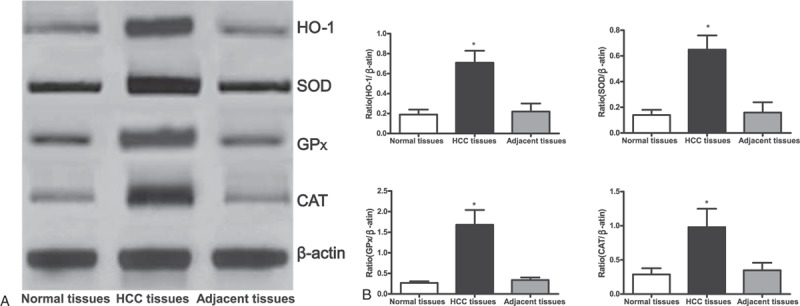
The expression of peroxiredoxins in normal tissue, HCC tissue, and adjacent cancer tissue. A = the expression of peroxiredoxins in normal tissue, HCC tissue, and adjacent cancer tissue, B = statistical analysis results of grayness ratio of HO-1, SOD, GPx, and CAT′ target bands and internal reference, ∗ = comparison with normal tissue, *P* <0.05, CAT = catalase, GPx = glutathione peroxidase, HCC = hepatocellular carcinoma, HO-1 = heme oxygenase-1, SOD = superoxide dismutase.

The results of Western blot showed that the expressions of heme oxygenase (HO)-1, glutathione peroxidase (GPx), superoxide dismutase (SOD), and catalase (CAT) in HCC tissue elevated significantly in comparison with those in adjacent cancer tissue and normal tissue (all *P* <0.05) (Figure 2). The results of RT-PCR showed that the mRNA expression level of HO-1, SOD, GPx, and CAT in HCC tissue significantly upregulated in comparison with those in adjacent cancer tissue and normal tissue (all *P* <0.05) (Table 3).

**TABLE 3:**
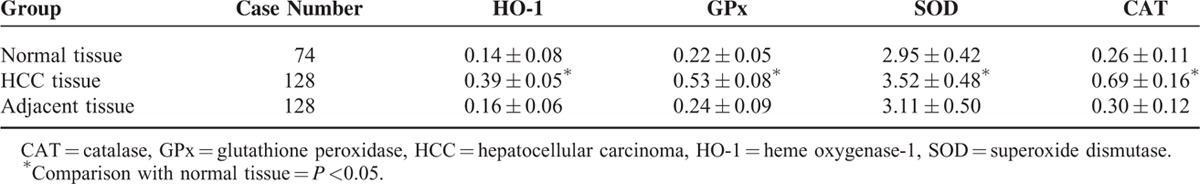
Testing Results of mRNA Expression Level in Normal Tissue, HCC Tissue, and Adjacent Tissue  
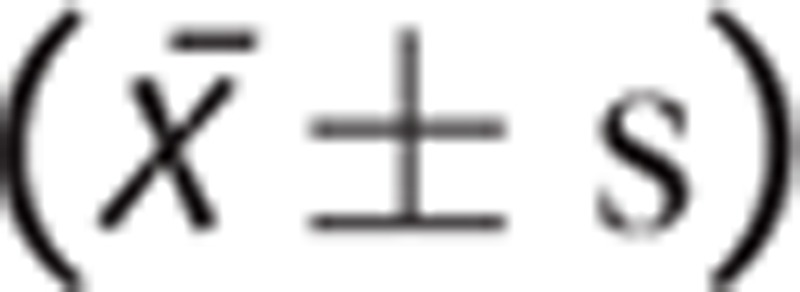

### Relationship between FOXO6 Expression And Clinicopathologic Features of HCC

The tumor size, tumor node metastasis (TNM) stage, AFP level, presence or absence of hepatitis B surface antigen (HbsAg), and differentiation degree were all related to FOXO6 expression level (*P* <0.05), but no significant associations between FOXO6 expression and age, sex, tumor number, violation of portal vein system, and lymph node metastasis were detected (all *P* >0.05) (Table 4).

**TABLE 4:**
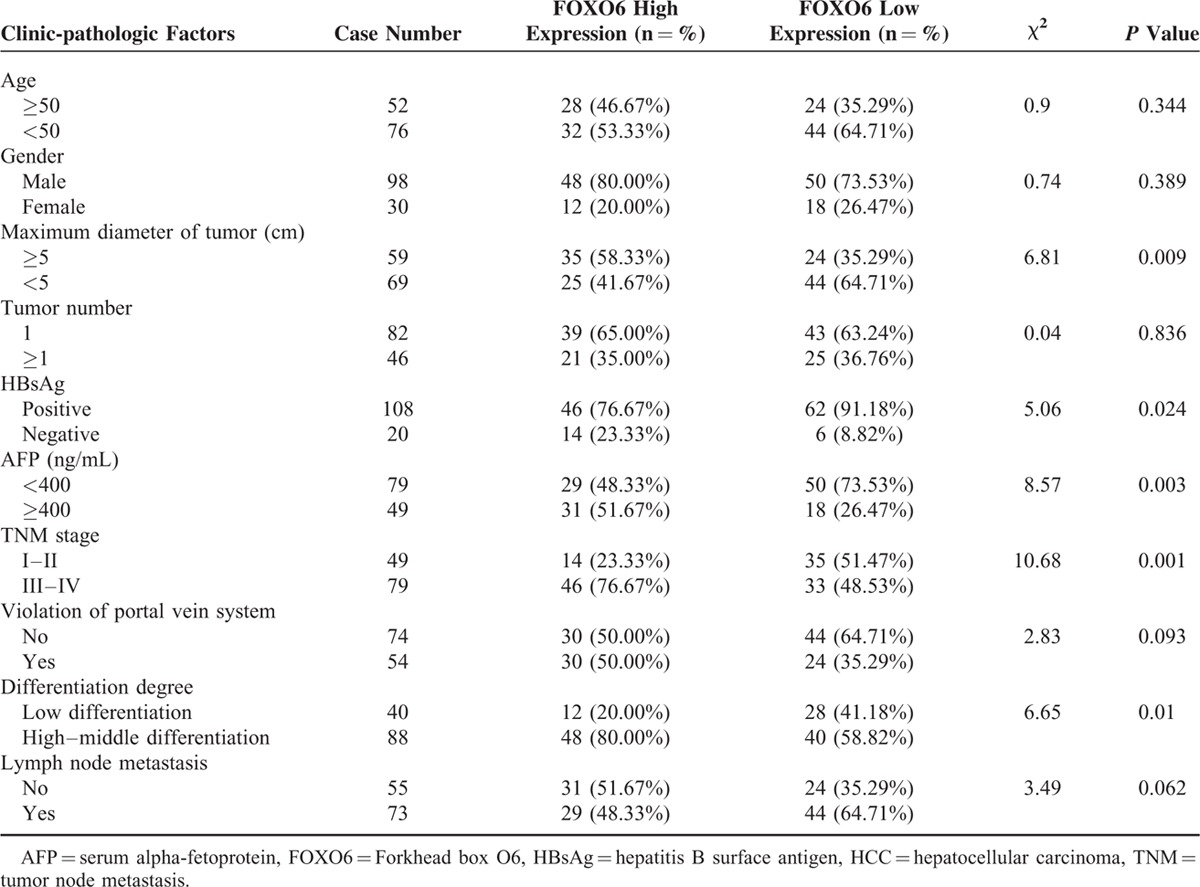
Relationship Between FOXO6 Expression and Clinicopathologic Features of HCC

### Prognosis Analysis

**FIGURE 3:**
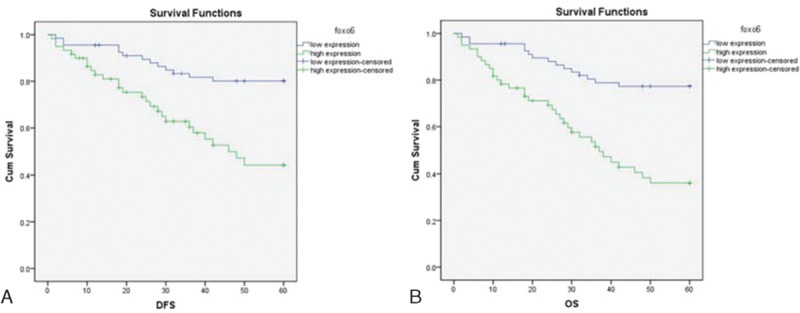
Kaplan–Meier survival curve of different FOXO6 expression levels. A = DFS survival curve, log-rank test shows *P* <0.05, B = OS survival curve, log-rank test shows *P* <0.05, DFS = disease free survival, FOXO6 = Forkhead box O6, OS = overall survival.

OS and DFS in patients with low FOXO6 expression were significantly longer than those in patients with high expression (both *P* <0.05). Age, sex, tumor size, tumor number, presence or absence of HbsAg, AFP expression, TNM stage, violation of portal vein system, degree of differentiation, lymph node metastasis, low FOXO6 expression, and high FOXO6 expression were considered as dependent variables in multivariate survival analysis using likelihood ratio (LR) method. Multivariate survival analysis showed that high FOXO6 expression, male, positive HBsAg, advanced TNM staging, high expression of AFP, and low degree of differentiation were the independent risk factors for the prognosis of HCC (all *P* <0.05) (Table 5 and Figure 3).

**TABLE 5:**
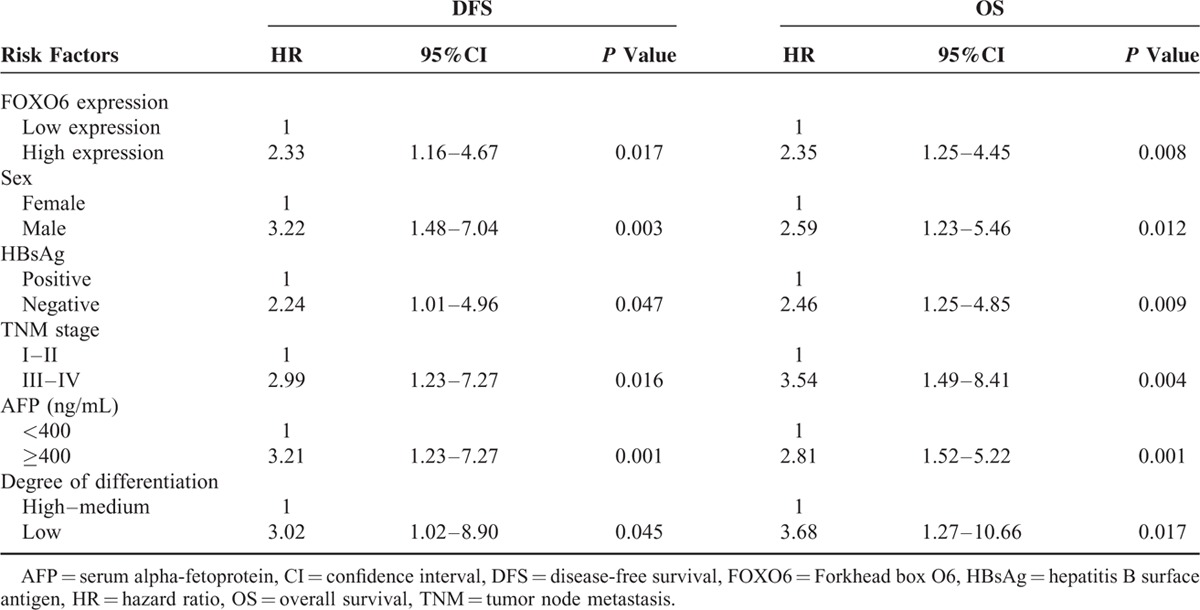
COX Risk Regression Model Analysis of Patients with Different FOXO6 Expression Level

### FOXO6 mRNA was Up-Regulated in 4 Cell Lines by RT-PCR

**FIGURE 4:**
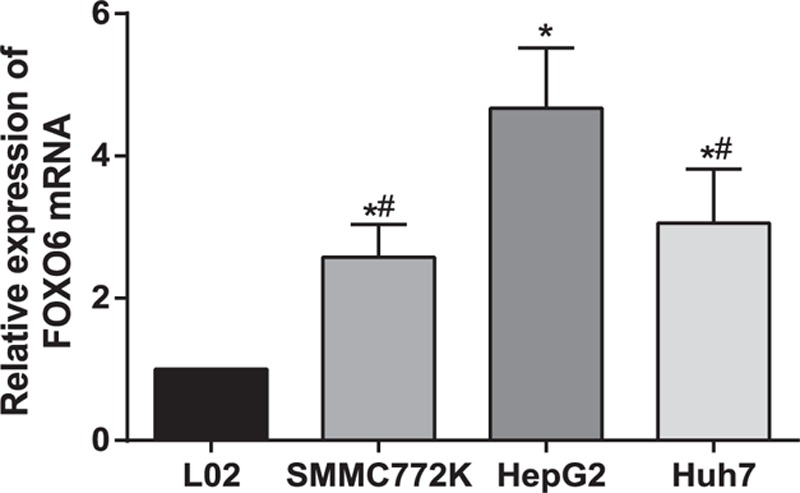
FOXO6 mRNA expressions in L02, SMMC772K, HepG2, and Huh7 cell lines by RT-PCR. ∗ = compared with L02 cell line, *P* <0.05, # = compared with HepG2 cell line, *P* <0.05, FOXO6 = Forkhead box O6, RT-PCR = reversed transcription polymerase chain reaction.

With β-actin as internal reference and L02 cell line as control, the level of FOXO6 mRNA was significantly higher in 3 cell lines SMMC772K, HepG2, and Huh7 than that of hepatocyte cell line L02 (*P* <0.01) by 2 to 4 times (Figure 4). Moreover, the expression of FOXO6 mRNA in HepG2 cell line was the highest (*P* <0.05) and there was no statistically significant difference between SMMC772K and Huh7 (*P* >0.05). As the expression of FOXO6 mRNA was the highest inn HepG2 and there was statistically significant difference with cell line L02, we therefore choose HepG2 as cell line performing RNA intervention in analysis.

### ROS and FOXO6 Level by RT-PCR

**FIGURE 5:**
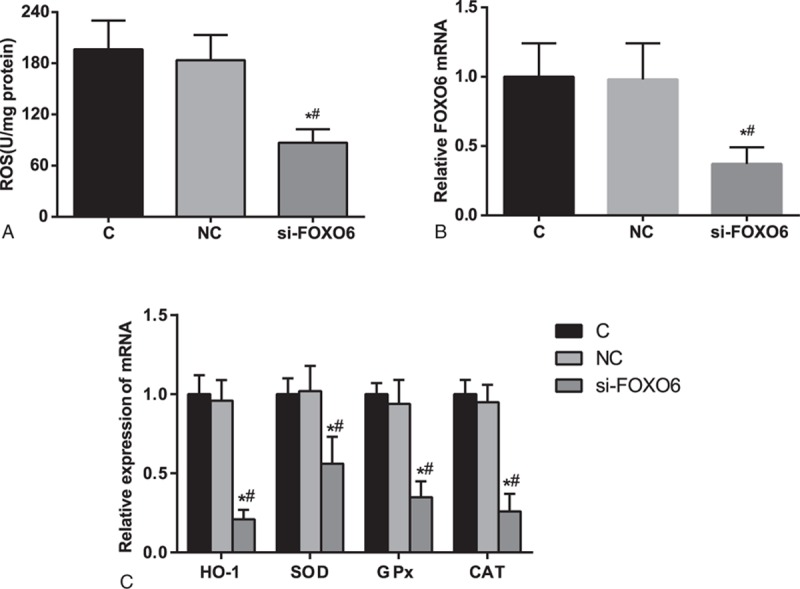
Detection on ROS level and FOXO6, HO-1, SOD, GPx, and CAT mRNA levels by RT-PCR. C = blank group, CAT = catalase, compared with the C group, *P* <0.05, # = compared with the NC group, *P* <0.05, FOXO6 = Forkhead box O6, GPx = glutathione peroxidase, HO-1 = heme oxygenase-1, NC = negative control group, ROS = reactive oxygen species. RT-PCR = reversed transcription polymerase chain reaction, si-FOXO6 = transfected group∗, SOD = superoxide dismutase.

The ROS level and RT-PCR results are shown in Figure 5. There is no obvious difference between C group and NC group in terms of ROS level, FOXO6, HO-1, SOD, GPx, and CAT mRNA levels (all *P* >0.05). Compared with the C group and NC group, ROS level, FOXO6, HO-1, SOD, GPx, and CAT mRNA levels in si-FOXO6 group were significantly decreased (*P* <0.05).

### Comparisons on Cell Proliferation

**FIGURE 6:**
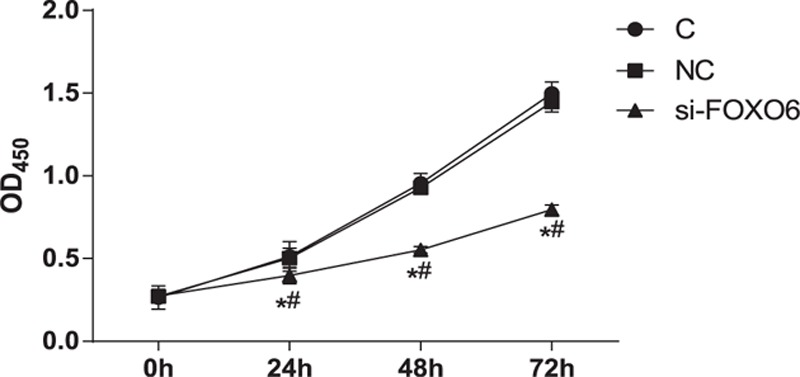
Comparison of cell proliferation among C, NC, and si-FOXO6 groups. C = blank group, NC = negative control group, si-FOXO6 = transfected group, ∗ = compared with the C group, *P* <0.05, # = compared with the NC group, *P* <0.05.

Compared with the C group, there was no significant difference in the NC group in terms of proliferation rate at times (all *P* >0.05). The comparison between C group and NC group indicated that the proliferation rate of the si-RNA group cells remarkably reduced (*P* <0.05) (Figure 6).

### Comparisons on Cell Cycle and Cell Apoptosis

**FIGURE 7:**
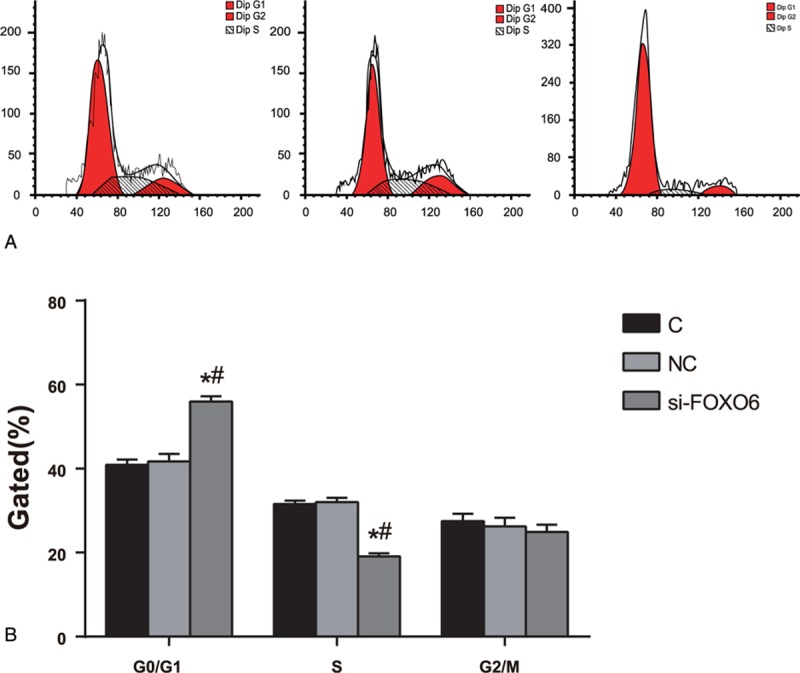
Comparisons on cell cycle among C, NC, and si-FOXO6 groups. A = period gram of flow cell, C = blank group, NC = negative control group, si-FOXO6 = transfected group, B = distribution chart of cell cycle, ∗ = compared with the C group, *P* <0.05, # = compared with the NC group, *P* <0.05.

**FIGURE 8:**
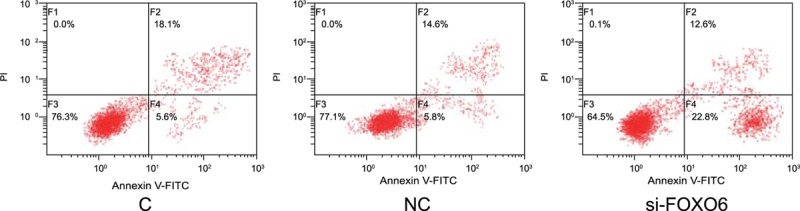
Comparisons on apoptosis rate of flow cell among C, NC, and si-FOXO6 groups. C = blank group, NC = negative control group, si-FOXO6 = transfected group.

According to the cell cycle detected by a flow cytometer, compared with the C group, there was no significant difference in G0/G1 period, S period, and G2/M period in the NC group (*P* >0.05). Compared with the C group and NC group, cells in G0/G1 period apparently increased in the si-FOXO6 group (*P* <0.05), and evidently decreased in S period (*P* <0.05) (Figure 7). Based on the apoptosis assay, the rates of early cell apoptosis in the C, NC, and NC groups were 5.6%, 8.3%, and 22.8%, respectively. Compared with the C group and NC group, apoptosis rate of si-FOXO6 cells increased notably (*P* <0.05). Meanwhile, there was no statistically significant difference between C group and NC group (*P* >0.05) (Figure 8).

### Expressions of p27 and Cyclin D1 Detected by Western Blot

**FIGURE 9:**
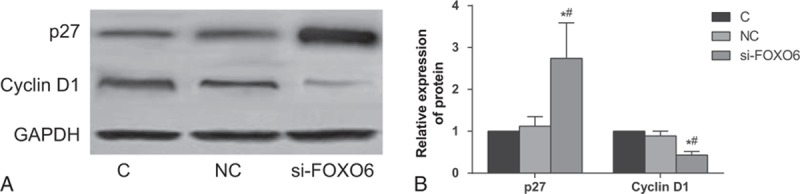
Comparisons on protein expressions of p27 and cyclin D1 among C, NC, and si-FOXO6 groups. C = blank group, NC = negative control group, si-FOXO6 = transfected group, A = relative expressions of p27 and cyclin D1 in contrast to GAPDH, B = histogram on relative expressions of p27 and cyclin D1 among C, NC, and si-FOXO6 groups.

Compared with the NC group, there was no obvious difference between the expression of p27 and cyclin D1 in the C group (*P* >0.05). Compared with the C group and NC group, the expression levels of p27 increased and cyclin D1 decreased apparently in the si-FOXO6 group (both *P* <0.05) (Figure 9).

## DISCUSSION

The morbidity and mortality of HCC is very high and its treatment effect is not satisfactory because most of primary HCCs have reach to middle-late stage at the initial diagnosis. Although AFP is widely accepted and implemented as a screening method for early stage of HCC, it still has limitations so that the study of new markers for diagnosis and prognosis of early HCC is significant.^18^ The study investigated the role of *FOXO6* in diagnosis and prognosis of cancer to explore the association between FOXO6 and the occurrence and prognosis of HCC.

In mammals, *FOXO* gene has functional specificity and has different expression levels in different tissues, for example, FOXO1 has significant expression in liver tissue and fat tissue, FOXO3 has significant expression in brain tissue, FOXO4 has significant expression in skeletal muscular tissue, and FOXO6 has expression in liver tissue.^19^ Although categorized in FoxO subfamily, FoxO6 differs from other FoxO members in being highly conservative and lacking PKB phosphorylation site of C-terminal.^20^ Our study found that the expressions of FOXO6, HO-1, GPx, SOD, and CAT in HCC tissue were significantly higher than that in adjacent cancer tissue and normal tissue. ROS levels in patients with high FOXO6 expression were significantly higher than those in patients with low FOXO6 expression which indicated that FOXO6 participated in the formation of HCC through oxidative stress. Liver is unique for its ability to regenerate and the key pathway for this process is Wnt/β-catenin signaling. Once this pathway was activated, β-catenin signaling drives the expression of target genes that are critical for cell cycle progression and contribute to initiation of the regeneration process.^21^ Therefore, aberrant activation of Wnt/β-catenin signaling may be an important subset for HCC occurrence. In addition, loss of β-catenin triggers oxidative stress.^22^ FOXOs, a β-catenin target gene, can bind to the 14–3–3 proteins to suppress their transcriptional activities once it was phosphorylated by Akt or SGK1; meanwhile, in the absence of phosphorylation, FOXOs can import to the nucleus and increase target gene expression.^23^ In addition, in neural stem/progenitor cells, ablation of FOXO impairs stem cell function by increasing ROS generation and reducing self-renewal capacity.^24^ In this regards, it is possible that FOXO6 regulates the ROS levels and contributes to the defects in the regeneration in HCC by interfering the Wnt/β-catenin signaling. Researchers have found that *FOXO* gene family could be used as markers of tumor diagnosis and prognosis since *FOXO* genes participated in the activities of cell cycle, apoptosis, and oxidative stress.^9^ Evidence suggested that OFRs played an important role in the occurrence of malignant tumors and oxidative stress during the process of HCC was important to the study of HCC treatment and prognosis.^25^ It is reported that when in cells occur oxidative stress, *FOXO* gene family can activate the expression of a series of target genes; therefore, cells are blocked in G2 stage and DNA is repaired, just as activated FOXO protein combined with gene promoters of coded manganese SOD and catalase produces stress resistance response.^26,27^ Presently, a study found that FOXO was activated to induce apoptosis and clear away cells that cannot be repaired when cells was damaged and couldn’t be repaired, and the activated *FOXO6* gene could regulate expression of peroxidase to achieve its oxidative stress.^28^

The study also found that TNM stage, AFP and tumor differentiation degree, male, positive HBsAg, and FOXO6 were independent risk factors affecting HCC prognosis. Our results also demonstrated that OS and DFS in patients with low FOXO6 expression were significantly longer than those in patients with high expression. The oxidative stress can damage the mechanism of cell signaling pathways and affect the function and expression of cells.^11^ ROS level in FOXO6 high expression patients’ tissue elevated significantly, resulting in protein damage, DNA damage, lipid peroxidation, and cell death.^29^ In many cell types, ROS can induce the activation of the FOXO transcription factors, which can mediate the effects of ROS through regulation of gene transcription.^30^ In addition, ROS leads to the generation of intracellular signals that stimulate inflammation and cell death. Therefore, it is reasonable to explain the phenomenon that high FOXO6 expression was an independent risk factor for prognosis of HCC. It is reported that FOXO6 can regulate the expression of proto-oncogenes; when FOXO6 has high expression this regulation function is activated, cancer cells increase rapidly; for instance, high expression of FOXO6 in gastric cancer cells will accelerate the proliferation of gastric cancer cells resulting in the progression of this disease.^31^

To further understand the possible role of FOXO6 in HCC, our study also conducted CCK-8 assay and flow cytometry to observe cell proliferation and cell cycle changes after silencing FOXO6 expression in HepG2 cells. Additionally, the expressions of P27 and cyclin D1 were also detected to explore the potential mechanism of cell arresting. Our results demonstrated that FOXO6 can remarkably arrest cell cycles and repress proliferation in HCC cells, which further supported that FOXO6 can significantly inhibit the development and deteriorate of HCC. Moreover, western blot further clarified that the si-FOXO6 group had an elevated expression of p27 and decreased cyclin D1 level. Progression on cell cycle is controlled by the sequential activation of Cdk–cyclin complexes which was negatively regulated by Cip/Kip protein family members, including p27, by inhibit their kinase activities at the G1/S- and G2/M-phases.^32^ Evidence showed that cytoplasmic p27 binds RhoA and modulates activation of the RhoA/ROCK cascade, thus plays an important role in cell apoptosis and migration, whereas oncogenic activation of the PI3K signaling pathway often results in cytoplasmic mis-localization of p27.^33^ As a member of the FOXO transcription factors, FOXO6 was involved in a variety of process, including cell cycle arrest, cell death, tumor suppression, metabolism as well as protection from oxidative stress.^34^ The vital part in these processes is the ability of FOXO to regulate, and be regulated by oxidative stress.^35^ Based on above-mentioned information, we hypothesized that FOXO6 may regulate cell proliferation and apoptosis by regulating p27 and cyclin D1 to activate the oncogenic PI3K signaling pathway, thus contributing to the tumor progression and deterioration. In conclusion, high expression of FOXO6 is closely related to the occurrence of HCC and its clinic-pathological features. Meanwhile, we also found that FOXO6 expression was the independent risk factor for HCC prognosis and it promised to be a marker for judging the deterioration and recurrence of HCC. However, the study still has some limitations. The functional mechanism of FOXO6 is various among different tissues and its specific role in the development of HCC is inconclusive. The present study paid much attention on the association between FOXO6 expression and the process of HCC occurrence, thus, no experiments were conducted to clarify the possible mechanism in HCC. Therefore, future studies are needed to investigate the specific function of FOXO6, its signaling pathway, and mechanism in HCC.
